# Metastatic myxoid liposarcoma of the brain: a case report and review of the literature

**DOI:** 10.2144/fsoa-2021-0077

**Published:** 2021-10-25

**Authors:** Baha'eddin A Muhsen, Ansam Ghzawi, Ahmad Salah Fares, Maysa Al-Hussaini, Samer Salah

**Affiliations:** 1 Department of Surgery, Division of Neurosurgery, King Hussein Cancer Center, Amman, Jordan; 2 Faculty of Medicine, Yarmouk University, Irbid, Jordan; 3 Faculty of medicine & Surgery, Jordan University of Science & Technology, Jordan; 4 Department of Pathology, King Hussein Cancer Center, Amman, Jordan; 5 Medical Oncology Department, King Hussein Cancer Center, Amman, Jordan

**Keywords:** brain metastasis, case report, MLS, MLS metastasis, myxoid liposarcoma, neurosurgery, soft-tissue tumor, stereotactic radiosurgery

## Abstract

**Aim:**

Myxoid liposarcoma (MLS) is a rare malignant soft-tissue neoplasm in which brain metastasis is still considered rare. We present a case of MLS brain metastasis and review of the literature.

**Case presentation:**

A 24-year-old male patient presented with headache, decreased level of consciousness and vomiting. He was treated for distal right thigh MLS 2-years ago. Imaging studies of the brain showed a left frontal intracranial mass. The patient underwent surgical resection followed by stereotactic radiosurgery. The histopathological study revealed metastatic myxoid/roundcell liposarcoma infiltrating the bone and peripheral margins.

**Conclusion:**

Treatment options for MLS usually includes surgical resection and adjuvant radiotherapy. A case-by-case based multidisciplinary approach is recommended for the management of similar cases.

Soft-tissue sarcomas are rare malignant tumors and account for 0.7–1% of all adult malignant tumors [1]. There are several subtypes of soft-tissue tumors; one of them being liposarcoma (LPS). This tumor is considered a soft-tissue sarcoma of mesenchymal origin and adipocytic differentiation. Myxoid liposarcoma (MLS) is the second most common type of LPS, as it roughly accounts for 20–30% of all LPS cases [2,3].

Another distinctive feature of MLS is its tendency to local recurrence and metastasis to the surrounding soft tissues and the skeleton. In contrast to other LPS subtypes which metastasize foremost to the lungs [3–5]. MLS metastasis to the brain is very rare with only few reports published in the literature with brain metastasis (BMs). BMs is seen in 20–40% of all cancer patients. Considering the tumors rarity and unexpected metastasis pattern, we present a case of MLS originating from the distal right thigh that metastasized to the brain.

## Case presentation

The patient is a 24-year old male who was diagnosed with right distal thigh MLS in 2018. He had wide local excision with neo-adjuvant radiation (50 Gy/25 fractions).

In February 2019, an MRI of the femur was obtained for restaging. The MRI did not show any signs of local tumor recurrence but was highly suspicious for an intramedullary enhancing osseous lesion involving the proximal right femur. A follow-up positron emission tomography (PET)/computed tomography (CT) was performed and revealed a mildly hypermetabolic intramedullary lesion within the proximal shaft of the right femur that was suspicious for malignancy.

In May 2019 the patient was given 2 cycles of chemotherapy (ifosfamide and doxorubicin), and later in the same month, an MRI for the thigh showed further progression in the proximal right femoral osseous lesion.

In June 2019, a biopsy was performed and was negative for any features of malignancy. Definitive radiotherapy (70 Gy/35 fractions) to the right femoral lesion was delivered to the patient by October 2019. The patient was then observed by regular follow up.

In November 2020, the patient presented to the emergency department with a decreased level of consciousness, headache and vomiting. The patient also had a gross lesion in his forehead which he neglected for a while. CT scan was performed and showed a left frontoparietal mass measuring 6 × 5.4 cm with vasogenic edema. The patient was admitted and treated conservatively without any surgical interventions as he was COVID-19 positive with mild symptoms. Chest, abdominal and pelvic CT scans were done and were negative respectively.

**Figure 1.**
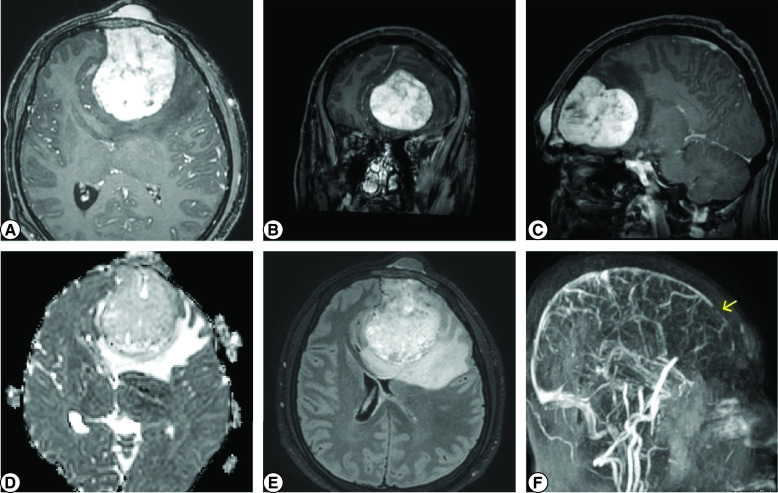
MRI of the brain with contrast. **(A)** Axial, **(B)** Coronal, and **(C)** Sagittal sequences showing large contrast enhancing left frontal mass with osseous infiltration, marked mass effect upon the adjacent brain, subfalcine herniation and marked compression of the frontal horns of the lateral ventricles seen. **(D)** ADC map sequence showing no restriction. **(E)** Flair sequence showing associated edema. **(F)** MRV showing invasion of the anterior part of SSS (arrow). ADC: Apparent diffusion coefficient; FLAIR: Fluid-attenuated inversion recovery; MRV: Magnetic resonance venography; SSS: Superior sagittal sinus.

**Figure 2.**
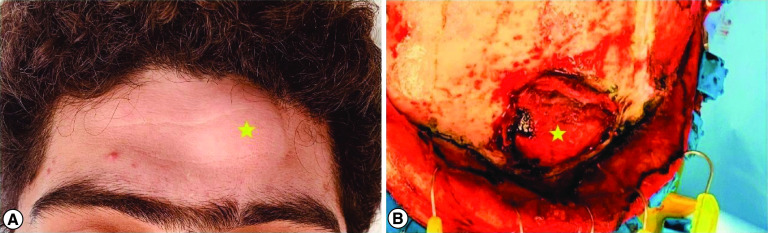
Clinical and operative view of the tumor. **(A)** Bump on the left side of forehead. **(B)** Operative view showing the tumor outpouching and involving the left frontal bone.

In December 2020, the patient was re-admitted with lethargy, drowsiness and decreased level of consciousness. Brain MRI showed a large contrast-enhancing left frontal intracranial mass with mass effect and uncal herniation (Figure 1). His Glasgow Coma Scale (GCS) score dropped during hospitalization to 6/15 and the pupil response was sluggish with left pupil dilation. He underwent left frontal craniotomy urgently with tumor resection including the affected bony involvement (Figure 2), except for a small part invading the superior sagittal sinus, followed by cranioplasty with of the methyl acrylic bone.

Histopathological analysis showed metastatic myxoid/round cell LPS. The tumor cells are negative for GFAP, S100, WT1, BCOR, SATB2, CD99 and cyclin D1.

**Figure 3.**
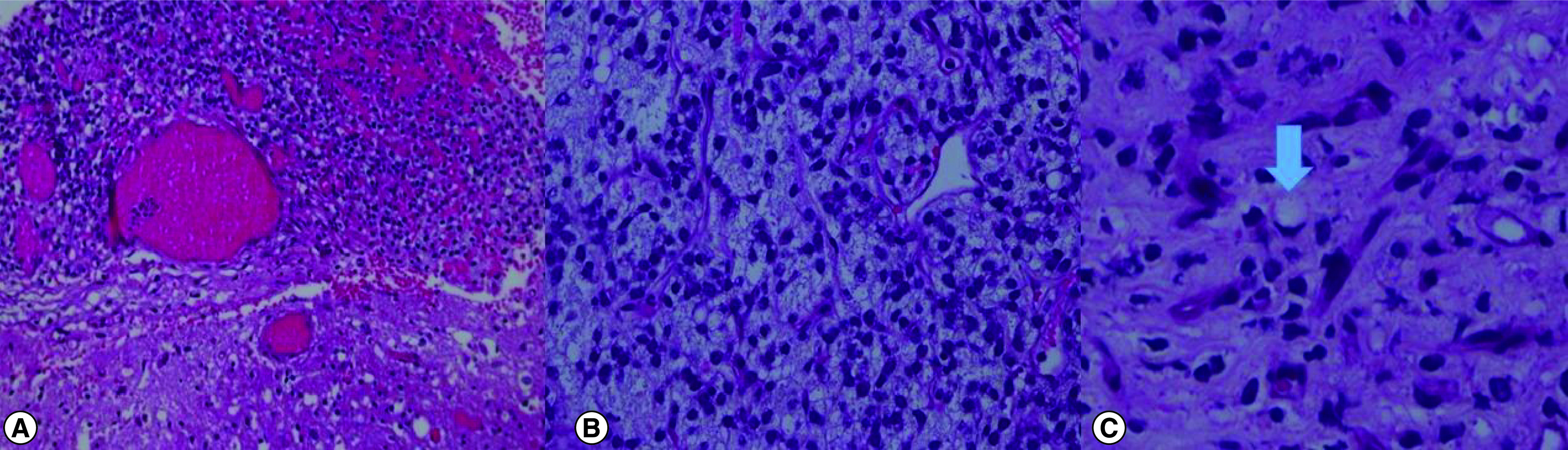
Histopathological slides. **(A)** Low power magnification with tumor (top half) infiltrating the brain tissue (lower half) (H&E × 4). **(B)** Low power magnification of the tumor composed of small round blue cells with chicken-wire vasculature characteristic of myxoid/round cell LPS (H&E × 10). **(C)** A univacuolated lipoblast is seen (arrow) (H&E × 40). H&E: Hematoxylin and eosin stain; LPS: Liposarcoma.

The tumor had infiltrated the skull bone and extended the peripheral margins (Figure 3). The case was discussed with the multidisciplinary clinic and the patient received stereotactic radiosurgery for the resection cavity. On week 6 follow up, there was no evidence of residual or tumor recurrence in the surgical bed. A new right parietal bone with soft-tissue component metastasis was treated with radiotherapy.

A follow-up staging CT scan showed a soft-tissue mass at the suprasternal notch related to manubrium sterni and an anterior peritoneal with involvement of linea alba metastatic deposit was found. Palliative external beam radiotherapy to the sternu was completed by January 2021.

**Figure 4.**
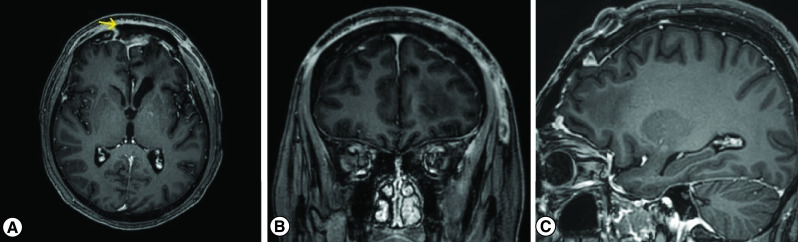
Postoperative MRI of the brain with contrast. **(A)** Axial, **(B)** Coronal and **(C)** Sagittal sequences showing en bloc tumor resection except the part inside the SSS with placing of an artificial bone-methylaacrylicacryltae (arrow). SSS: Superior sagittal sinus.

Four months later, follow-up brain MRI revealed a right occipital condyle lesion, mostly correlating with osseous metastasis. There was no evidence of recurrence at the surgical bed (Figure 4), and no significant change in the previously treated right parietal lesion. The patient was treated with radiotherapy for the right occipital condyle lesion.

The patient was observed by regular follow-up imaging. On his 6th month follow up, he was alive and all lesions were stable except the peritoneal lesion was slightly progressive. He was placed on single-agent dacarbazine.

## Discussion

Soft-tissue sarcomas are rare malignant tumors, they account for 0.7–1% of all adult malignant tumors [1]. There are several subtypes of soft-tissue tumors and among them is LPS, which is considered a soft-tissue sarcoma of mesenchymal origin and adipocytic differentiation. It is reported to be the second most common soft-tissue sarcoma as it is responsible for 15–20% of all soft-tissue sarcomas, with peak incidence between 40 and 50 years old [6,7].

According to both the molecular and clinicopathologic dissimilarities, there are five subtypes of LPS acknowledged by the WHO in the latest update of LPS classification published in 2020: atypical lipomatous tumor also known as well-differentiated LPS, dedifferentiated LPS, MLS, pleomorphic LPS and myxoid pleomorphic LPS [6].

MLS is the second most common type of LPS, as it roughly represents 20–30% of all LPS cases [2,6].

There are multiple distinctive features of MLS including its clinical presentation, metastatic patterns, genomic and histopathological findings that help to segregate it from the other subtypes of LPS [8].

MLS is painless, which leads to a large presenting tumor usually over 10 cm. It typically occurs in young adults and peaks between 30 and 50 years [9]. MLS exclusively appears in children and adolescence and is considered the commonest subtype of LPS in these age groups [3].

Upon reviewing the literature, several studies reported the proclivity of MLS to present most commonly as slow growing, deep-seated in the soft tissues of the lower extremities, with two-thirds of the cases arising within the thighs.

Moreover, another distinctive feature of MLS is its tendency to local recurrence and extra pulmonary metastasis such as the soft tissues and the skeleton, in contrast to other LPS subtypes which metastasize foremost to the lungs [4,9,10].

Several studies reported different percentages of distant metastasis; however, almost all studies reported MLS to have an unusual metastatic pattern. For example, Estourgie *et al.* reported that 13 out of 14 patients with distant metastasis had extrapulmonary lesions. The retroperitoneum and the spine were the most common sites, respectively [5]. It is rare for the brain to be the first site of metastasis in MLS [11].

Our patient’s MLS first metastasized to the brain; however, there are only few cases published in the literature with brain as the first site of metastasis [12–19] Summarized in (Table 1).

**Table 1.** Summary of the liposarcoma cases metastasizing primarily to the brain.

Study (year)	Age (years)/sex	Primary site	Histology	Diagnosis of brain metastasis	Duration to brain metastasis	Site of brain metastasis	Treatment	Is the brain the first site of metastasis	Ref.
Bitoh *et al.* (1985)	49/F	Mesentery	Myxoid	Autopsy	–	Suprasellar	–	–	[12]
Haft *et al.* (1988)	52/M	Thigh	Myxoid	CT	18 years	Temporoparietal lobe	Surgical resection, chemotherapy	Yes	[13]
Paraf *et al.* (1990)	27/M	Heart	Myxoid	CT	–	Parietal lobe	Surgical resection	Yes	[14]
Can *et al.* (1993)	22/M	Pericardium	Round cell	Autopsy	–	Multiple	–	–	[15]
Fitzpatrick *et al.* (1999)	74/F	Thigh	Myxoid	CT	2 years	Parietal lobe	Surgical resection	Yes	[16]
Utsunomiya *et al.* (1999)	44/M	Thigh	Myxoid	CT/MRI	6 years	Left frontal lobe	Surgical resection	No	[17]
Kumar *et al.* (2000)	73/F	Thigh	Myxoid	CT, MRI	Over 1 year	Temporoparietal lobe	Surgical resection	Yes	[18]
Zagzoog *et al.* (2017)	43/F	Scapula	Myxoid	CT, MRI	2 years	Sellar and parasellar regions	Surgical resection	Yes	[19]
Current case	24/M	Thigh	Myxoid	CT, MRI	2 years	Left frontal lobe	Surgical resection, radiotherapy, chemotherapy	Yes	

Adm: On admission; CT: Computed tomography; F: Female; M: Male.

There are also some reported cases in which the brain was the primary origin of the patients LPS The last of them was reported by Watanabe *et al.* in 2018 with a summary of the previous literature [20]. To confirm, the diagnosis of MLS mostly depends on imaging (CT or MRI) and biopsy. Although there is a debate about the type of biopsy – core biopsy is more cost effective and is safer to perform than an open biopsy [21].

The management of LPS can differ according to the subtype, site presentation and whether it is a primary disease or if it is a recurrence with local or distant metastasis. It is suggested that the evaluation should be conducted by a multidisciplinary team.

Surgery, radiotherapy and chemotherapy are usually included in the treatment plan in a varying extent [22]. MLS have a high risk to metastasize and classically metastasis occurs with no symptoms until later in the disease. So, multiple tools, like the Memorial Sloan–Kettering Nomogram were established to calculate the risk of metastasis [23].

Some studies suggest that in patients with small or mildly symptomatic BMs, there can be a major advantage of gamma knife radiosurgery over surgery is that it is less invasive and not associated with significant recovery time so that systemic therapy can be administered sooner [24,25].

MLS must be considered for LPS patients who present with neurological manifestations early on. Early management can prevent further progression and improve the patient’s prognosis accordingly. A case-by-case based multidisciplinary approach is still recommended for the management of such patients to achieve the most favorable outcomes.

## Conclusion

MLS is a rare soft-tissue tumor that has a distinct presentation and metastatic pattern. It is usually treated with surgical resection followed by radiotherapy, and it tends to be located deep within the thigh. Very few reports have been published in the literature regarding metastatic LPS. Brain metastasis is rare and might be challenging, especially in cases of multiple cranial metastasis. Surgical resection with radiotherapy and adjuvant chemotherapy is the management option of choice; however, a case-by-case based multidisciplinary approach is recommended for the management of patients to achieve the most favorable outcomes.
